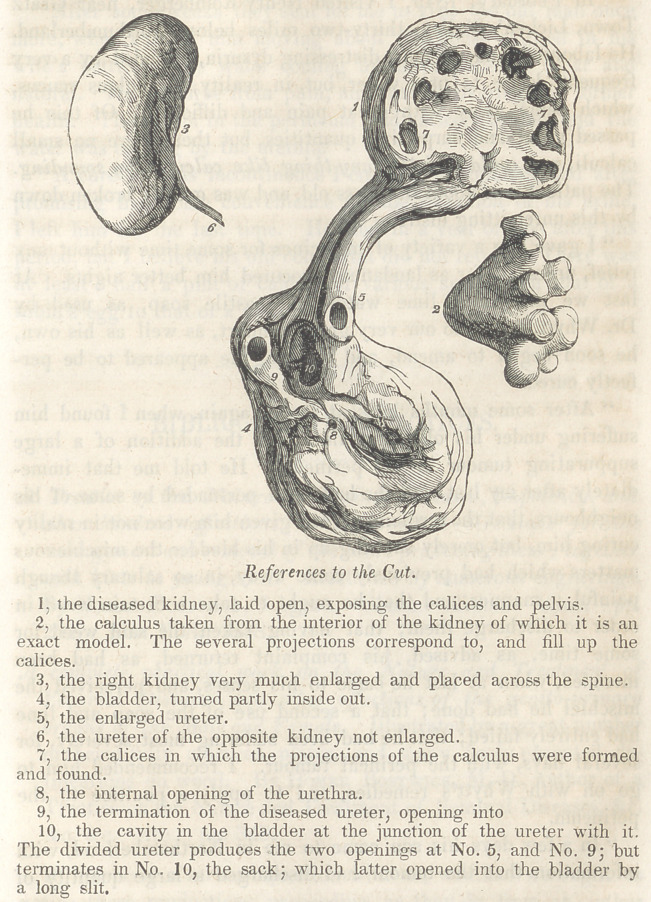# An Essay on Pyelitis Calculosa, or Stone in the Kidney

**Published:** 1848-11

**Authors:** James Bryan

**Affiliations:** Philadelphia, Member of the American Medical Association, Professor of Surgery in Geneva Medical College, N. Y., &c., &c.


*An Essay on Pyelitis Calculosa, or Stone in the Kidney. Translated from the French of P. Rayer, Physician to the Hospital de la Charité, &c., &c., &c. With notes and additional cases.* By James Bryan, M. D., of Philadelphia, Member of the American Medical Association, Professor of Surgery in Geneva Medical College, N. Y., &c. &c.

(Concluded.)

After stating the value of these symptoms (pain in the renal region, the presence of mucus, blood or pus in the urine) as signs or pyelitis calculosa, I proceed to state the distinctive characters of *renal tumours* formed by the accumulation of pus in the pelvis. I shall insist upon the diagnosis of these tumours in a special manner, because of the operations which they demand; operations which may be useless or promptly mortal if they are practised on other tumours, sometimes observed in this region.

Tumours which may be confounded with those which are consecutive to chronic pyelitis calculosa, are, on the left side, all those which result from a morbid enlargement of the spleen; on the the right side, tumours of the liver and of the gall bladder: on both sides different renal tumours of another nature, (hydro-nephrosis hemorrhages in the pelvis, cancerous or tuberculous kidneys: kidneys containing acephalocysts, &c.,) extra renal abcesses, either idiopathic or consecutive to perforations of the kidney, or of the colon, or of the end of the cœcum: abcesses from congestion following caries of the spinal vertebræ; tumours formed by supra-renal aneurisms of the aorta: encysted tumours containing various fluids or acephalocysts: finally stercoraceous tumours produced by the accumulation of fœcal matters in the colon and in the cœcum.

1. Of all the tumours which may be confounded with those produced by an accumulation of pus in the pelvis, the *hydro-nephritic* are, without doubt, those nearest, both from their form, their situation, and from the fluctuation felt throughout their extent. In the two cases, the tumour formed by the enlarged kidney is usually nodular, fluctuating, flat on percussion, and accompanied by an enlargement of the lumbar region. But the tumorus formed by the accumulation of pus in the cavity of the pelvis or calices, are the seat of more or less acute pain at one time or other; they are often accompanied by febrile action; and if they are indolent, become painful on pressure or movement of the body. In hydro-nephrosis on the other hand, the tumour is indolent, and only inconvenient on account of its size. Finally, when the communication between the pouch formed by the distended kidney and the ureter is not completely interrupted, the urine in the pyelitic tumour is purulent and opaque, while in the hydro-nephritic case it is commonly transparent or only obscured by mucus.

2. It is difficult to distinguish a tumour formed of a purulent collection in the pelvis from abcesses situated in the cellular tissue surrounding the kidney, (peri-nephite,) whether these abscesses supervene upon a contusion, or on the passage of pus or urine through renal fistulæ following an inflammation of the pelvis or of the kidney. For the rest, it is to be observed, in a tumour formed by a collection of pus in the cavity of the pelvis, that the fluctuation is deeper in the lumbar region than that of an abscess about the kidney, and this is nearly always followed or accompanied by œdema of the subcutaneous tissue of the loins, which I have never seen in the former case unaccompanied by the latter; nearly always, abcesses situated between the posterior surface of the kidney and the lumbar muscles, terminate by elevating the skin; and by the application of one hand on the anterior surface of the abdomen, and the other on the lumbar region, the fluctuation is more sensible than when the pus is only in the pelvis and calices. The passage of pus with the urine will decide the question, but even this sign is wanting when the passages are entirely obstructed.

3. It is more difficult to distinguish in the complex cases where there is at once pus in the pelvis and calices and outside of the kidney. At the same time, after the formation of a purulent collection in the cavity of the pelvis, there occurs at a later period an acute pain in the back part of the tumour, a swelling under the skin; the succession of these symptoms will indicate fistula renales and an abcess of the kidney. But there are cases in which this order of succession of these accidents has not been observed, or the characteristic marks of the lumbar tumour are absent; here the diagnosis is uncertain, unless the patient have suffered retention of urine, bloody urine, or nephritic colics. In such a case, when once it is decided that the lumbar tumour is not formed of blood, the surest mode of making out the diagnosis clearly, and of preventing the collection of a fluid in the abdominal cavity, or at least the extension of the abscess, is to open the tumour. The character of the fluid from the tumour will clear away all doubt. A fœtid odour, like that from stercoraceous abscess may arise from purulent deposits around the kidney or colon without perforation of this intestine. On the other hand, if a stercoraceous odour does not exclude the idea of an abscess about the kidney, with or without communication with the interior of the pelvis, we must remember that purulent collections formed in the interior of the kidney opening externally, have in many cases no sensible urinary odour. Flakes perceived by the fingers in the abscess do not necessarily authorize us to consider the contents as urinous. I have seen, indeed, tuberculous abscesses of this region present such filaments and a species of organic attritus. But if the diagnosis is not entirely satisfactory at the time of opening the abscess, the after symptoms will make all clear.

4. In stercoraceous abscesses, even fœcal matters pass out through the fistula; percussion and pressure on the colon favour their exit. Worms and solid food may pass out at the wound, &c.; pus is sometimes evacuated with the fœces at stool.

In extra renal abscesses, without fistula, neither fœcal matters nor urine pass through the wound. In purulent collections following pyelitis calculosa, urine and sometimes one or more calculi, after the evacuation of pus, pass off.

5. Abscesses around the kidney, consecutive to abscess of the liver, cannot by means of the pus be distinguished from pyelitis and other abscesses of this region. The colour of the lees of wine which some authors attribute to the pus of hepatic abscesses, is rarely seen. The source of these abscesses may be perhaps found in the functional derangements of the liver.

6. Abscesses from congestion, supervening on caries of the vertebral column, or on tubercular affection of the spine, may be distinguished from distension of the kidney by pus, in as much as they are almost always accompanied with projections of the spinous processes of the vertebræ, and more frequently by paralysis of the inferior extremities, the bladder and the rectum; besides, these kinds of abscesses, nearly always placed behind the ascending or descending colon, form in the abdomen a tumour nearer the vertebral column, more elongated, and directed obliquely from the spine towards the crural arch. I have also seen caries of the ossa ilia produce purulent collections which rose towards the renal region; they differ from purulent tumours of the kidneys in being smoother on their surface. Nevertheless, these abscesses and those connected with caries of the spine, or with tubercles of the lower spinal vertebræ, sometimes form lumbar tumours, which resemble extra-renal abscesses, following open renal fistulæ.

7. Blood, after a contusion of the lumbar region, has been known to be suffused in the cavity of the renal pelvis, so as to enlarge both this and the kidney. In this case, which is very rare, the cause of this disease and the habitual discharge of blood without mixture with the urine, will lead us to a true diagnosis.

8. The serous or urinary cysts of the kidneys are rarely sufficiently numerous to imitate a collection of pus. But an acephalocystic cyst in the kidney may simulate a tumour produced by pus in the pelvis. The rubbing, proper to acephalocystic tumours, can be perceived in a small number of cases only, and the evacuation of the acephalocysts with the urine, a symptom which clears all doubt in the case, takes place only after the rupture of these cysts in the pelvis.

9. Tubercular kidneys are rarely large enough to simulate a purulent distension of the pelvis and kidney. The sensation of hardness presented by a kidney filled with tubercles, is very different from that of the renal pouches filled with pus. Ramolissement of the tubercular kidneys may be followed by a fusiform abscess toward the crural arch, not only difficult to distinguish from a purulent collection in the pelvis and vicinity of the kidney, but also from abscesses of congestion supervening on caries of the vertebræ.

10. The cancerous kidney may be so enlarged that it will weigh several pounds, and form a tumour in the lumbar region. This tumour may present an obscure fluctuation, while the greater part of it is composed of blood; but frequent hæmaturias with the external characteristics of cancerous diathesis, will point out the difference between these tumours and those composed of pus in the pelvis.

11. Tumours formed by the capsulæ renales distended with blood or other fluids, are rarely large enough to simulate purulent tumours of the pelvis and calices. For the rest, these tumours are neither preceded nor accompanied by pus in the urine.

12. Aneurisms of the descending aorta have been known to simulate abscesses, or a purulent collection in the pelvis or calices. Pulsations synchronous with those of the pulse, a sensation of expansion perceived by the hand, and in a large number of cases the blowing sound perceived on auscultation, will decide the case.

13. Enlargement of the spleen cannot be mistaken for the left kidney distended with pus. The tumour formed by the spleen is higher up in the region of the large extremities of the stomach, and projects more forwards than renal tumours; besides, enlargements of the spleen are found nearly always in persons who have long suffered with intermittent fevers; flatness on percussion is perceived from the superior part of the left hypochondriac region towards the umbilicus. The spleen is usually a solid, perceptible to the touch, either when we press on the anterior parietes of the abdomen, the thighs being flexed on the hips, or when we rub with the hand the surface of the tumour.

14. A morbid enlargement of the left lobe of the liver or of its base, in consequence of a serous or sero-purulent deposit in the right pleura, may simulate a tumour formed of pus on the right kidney; and that the more readily in as much as renal tumours always terminate by contracting adhesions to the liver.

15. Acephalocystic cysts of the liver may, to a certain extent, simulate a dilated right kidney adherent to the liver; but besides that these acephalocystic tumours of the liver are more superficial, and afford to the finger a particular rubbing sensation, they are not accompanied with purulent or bloody urine. The same remark may be made in reference to tumours of the gall bladder. These tumours, in elongating, remain pyriform, and do not project behind the loins, except when they attain considerable size.

16. I have seen tumours connected with the ovaries, projecting into the lumbar region; but these tumours more moveable than renal tumours, may generally be pressed into the hypogastric region.

17. Cysts of the cellular tissue of the abdomen and of extra-uterine pregnancies are very rare occurrences, and bear but faint analogies to tumours of the kidneys.

18. It is not always possible at the first examination to distinguish stercoraceous tumours, occasioned by the accumulation of fœces in the ascending or descending colon, from renal tumours, when, as in the latter cases, the lumbar regions are flat on percussion, at least on the lateral portion, when these matters are collected in greater or lesser masses, a morbid sensibility of the intestine corresponding to the kidney is found; and when seen in individuals who have formerly had functional diseases of the urinary organs, there certainly is some uncertainty in forming the diagnosis. At the same time the tumours formed in the ascending or descending colon by obstructed fœcal matters, are usually more cylindrical than renal tumours. On the right side, stercoraceous tumours often extend towards the cœcum, which sounds flat in some points, and sonorous in others, where it is distended with gas. On the left side the fœcal tumour extends to the iliac fossa, and towards the excavation of the pelvis. We often at the same time perceive that the transverse colon contains hard and solid fœces. For the rest, fœcal tumours are more common on the right than on the left side; and when they are painful on pressure or to the touch, it is more common in front than back; which is the reverse of what is observed in most cases in morbid dilatations of the kidneys, when there is not inflammation of the corresponding portion of the peritoneum: finally, fœcal tumours disappear after free purgation.

Having decided that a lumbar tumour is formed by the accumulation of a quantity of pus in the pelvis and calices, and perhaps at the same time in the extra-renal tissue, we must look for the cause of such a collection. For, before proceeding to the treatment, we must decide whether the suppuration and distension of the pelvis have followed a retention of urine, occasioned itself by a stricture of the urethra, a tumefaction of the prostate, or a paralysis of the bladder; or, indeed, whether the distension of the kidney is the consequence of an occlusion of the ureter, produced by an excrescence or by a calculus in its passage, by a tumour in its vicinity, or by calculi formed in the pelvis or calices.

The diagnosis of primitive diseases, antecedent to the pyelitis, makes it necessary to explore the urethra, prostate, and bladder, and to examine with great care the different regions of the abdomen. And this is not all, the diagnosis will be incomplete unless we proceed with the same care to the examination of the secondary diseases.

Of all the complications of pyelitis calculosa, the most frequent is nephritis. In chronic pyelitis, the progressive atrophy of the substance of the kidney renders this complication more rare and less important. We then come in the order of their frequency to diseases of the bladder, the prostate, and the urethra, producing retention and consequent disease of the kidney.

*Prognosis.*—However painful pyelitis calculosa is, in its first stage, it is generally not serious when one kidney alone is affected. The prognosis, on the contrary, in the second stage, and especially in the third and fourth stages of the chronic form, even where the patients preserve the appearance of health, and the urine is not altered except by the augmentation of mucus, is always dangerous. For supposing for an instant that the pelvis becomes accustomed, so to speak, to the presence of a renal calculus, (I have collected several examples of this kind) this foreign body, in its developement, will terminate by inducing the total atrophy of the kidney, or a complete retention of pus and urine in this organ; and should the other kidney become influenced, or the ureter become obstructed, even temporarily, by a calculus, rapid death may be consequence.

In pyelitis calculosa with purulent secretion, the prognosis, dangerous when one kidney is affected, is much more so when both are diseased. We not only expect a progressive atrophy of the kidney and the consequent diminution of the secretion, but other dangers follow the suppuration of the interior of the pelvis and other parts of the kidney, viz: the possibility of a perforation or an absorption of pus, peritonitis, pulmonary phthisis, &c. At the same time I have already remarked that in fortunate cases, the other kidney remains sound, but enlarges in proportion to the increased function assumed by the loss of the first.

The prognosis is also more or less grave, according as the pus is retained in the pelvis or is discharged into the bladder. In pregnant women the disease is a cause of abortion and premature labour. The formation of lumbar fistula may be considered favourable. Communications with the liver, lungs, or intestines, &c. are generally mortal: death is sudden when a collection forms in the cavity of the peritoneum.

A complete occlusion of the ureter, preventing the passage of the purulent urine into the bladder, if prolonged, is dangerous, unless relief is obtained by means of a fistula. When these collections of pus are followed by blind fistulæ, the larger abscesses which form around the kidney are much more dangerous than simple dilatations of the pelvis and calices.

The prognosis in this disease is also rendered dangerous by concomitant diseases of the urinary or other organs.

*Treatment.*—At the beginning of pyelitis, and in its first stage, the nephritic pains are relieved by warm baths and mucilaginous drinks, flaxseed tea, emulsions, small beer, and laudanum, &c.; by leeches, or better by cups applied to the painful part.

When the pain is very acute and accompanied with suppression of urine, violent efforts at vomiting with a tendency to syncope, ether drinks, fomentations of assafœtida, or of the leaves of henbane, frictions of the tinctures of camphor and opium, or better, fumigations of ether, will fulfil the first indication, that of relieving the spasms, nausea and syncope.

Generally these symptoms cease after a few hours, either by the expulsion of the gravel or by its passage into the bladder. In such cases cold is sometimes useful; the patient being stripped, and his feet placed on the pavement. This practice has often been followed by the expulsion of the gravel or by the re-establishment of the urinary secretion. This expulsion is also favoured by the application of dry cups in the course of the ureter or on the perineum.

When the primary symptoms have yielded, if the gravel be not expelled, if the urine becomes charged with mucus and the case takes on the characteristics of the second or third stage, we must explore the bladder: and if a small calculus is found, it should be extracted or broken, or its expulsion should be promoted by causing the patient to drink large quantities of spring or mineral water.

Should a calculus be found in and obstructing the ureter, (indicated by the locality of the pain and diminution of the urine, &c.) we are recommended to induce vomiting, coughing and sneezing, and to induce the patient to undergo rapid movements; in this way to subject the body to concussions, in order to favour the passage of the gravel into the bladder. On this subject I would remark that I have rarely seen the expulsion of the gravel produced by either spontaneous or induced vomiting: and the patient suffers too much pain for us to induce either coughing or sneezing.

As for the cases in which the ureters are obstructed by calculi, the practice is absolutely useless and dangerous. When gravel or calculi remain fixed in the calices, the arch of the pelvis or the ureter, inflammatory symptoms thus induced may be relieved by warm baths, flaxseed tea, mineral water or wet cupping.

The sitting posture, continued for several hours, nearly always produces pain. This is a fact observed in sedentary men, engravers and others, who sit long inclined forwards. On the other hand, the recumbent posture on one side or the other, with the thighs flexed relieves the pain.

Patients should abstain from sexual intercourse. I have often found when the pain has resisted cups, that flying blisters applied to the renal region will relieve it.

We may for a time, by our treatment, relieve the inflammatory symptoms induced by the presence of gravel in the pelvis or calices; but they will always or nearly always remain charged with mucus or muco-pus. With the view of diminishing this secretion, oil of turpentine, in graduated doses, from twelve drops to a drachm per diem, for an adult, has been used. I have also used the balsam of copaiba, the prepared turpentine, a tisan of the buds of the fir tree, cubebs, &c., and I am bound to say in some cases the mucus or muco-pus has been diminished, the exacerbations of renal pain have also been less frequent. In fact, I think that the milk of anise and flaxseed water are the drinks which should habitually be used by the patient in a great majority of cases.

The second indication is the physico-chemical treatment of the renal gravel or calculi. Certain drinks which may be taken in large quantities, such as spring water, and the water of Contrexville, act mechanically in washing the calculi, carrying the smaller gravel through the ureters, and facilitating, by frequent emissions of urine, the expulsion of these bodies. The action of these drinks on the renal calculi, properly so called, is confined to so narrow a space, that in spite of all that can be said, little is to be expected from them. We must not expect too much either from the chemical action of certain substances on gravel and calculi of different compositions. Doubtless experience teaches that alkaline drinks are generally favorable in the uric acid calculi, and in pyelitis produced by concretions; but attentive observation on the action of these drinks, proves also that in gout, (a pathological condition in which the uric acid gravel and calculi are most commonly generated,) the uric acid deposit persists after many cures by alkaline waters; and that also the continued use of these is followed by serious inconvenience.

When it is found on examination of the urine that the gravel or calculi are composed chiefly of the phosphates (at least in their exterior layers,) chemistry, properly so called, presents to us but few resources. Generally, we advise the use of carbonic acid drinks; but it is certain that these very seldom render phosphatic urine clear and transparent. For the rest, a considerable experience has taught me that an alkaline troubled urine is, if not always, at least nearly always, the result of chronic inflammation of the kidneys. We cannot expect to modify such urines by either vegetable or mineral acids; but may do it by the treatment recommended for chronic nephritis. (See chapters on oxalic, phosphatic and cystic gravel.)

In the fourth stage of pyelitis calculosa, where urine and pus are collected, and accumulated in the pelvis and calices, in a manner to form a tumour in the lumbar region, we are directed, in order to favour the passage of the pus through the ureter, to attempt the displacement of the calculus by pressure or concussion of the trunk, by long rides on horseback or in a carriage. But to suppose that a calculus can be displaced in this way, so as to allow of the passage of pus along the ureter, is to suppose that a foreign body produces no inflammation in the ureter closing its diameter. On the other hand, pressure on the renal tumour when it is already painful, would be mischievous. By this treatment we would excite inflammation, and perhaps rupture the tumour into the cavity of the peritoneum, or into the extra-peritoneal cellular tissue.

When the pus passes in small quantity every day from the pelvis into the ureter and bladder, either along a furrow which the pus and urine have formed on the calculus, or between the calculus and the ureter; if the suppuration of the pelvis be not great, and does not produce hectic fever; if the renal tumour is not painful; if, on becoming full, it easily evacuates itself by the ureter; if the neighbouring parts, the peritoneum, the liver, the spleen, present no signs of present or past inflammation, if the patient has no diarrhœa, and is not materially debilitated, we should attempt to prevent the establishment of acute inflammation in the tumour or neighbouring parts, by rest, by the daily use of baths, by topical emollients and by a well regulated diet. We must combat by timely venesections proportioned to the intensity of the disease, and the strength of the patient, the inflammation as it is observed to occur in these tumours. Other circumstances, such as the advanced age of the patient, serious lesions of the bladder, of the uterus, &c., may reduce the treatment to one purely palliative.

But there are other conditions in which nephrotomy appears justifiable, or at least in which the opening of the pouch of the pelvis and calices, and the extra renal cellular tumour, should not be deferred.

Thus, when such a tumour exists in an individual otherwise well organized; if it is habitually painful, in spite of the use of mucilaginous and oily drinks, baths and leeches, if the fever is continual or with nocturnal paroxysms; if the stomach and intestines are continually in a morbid condition; if the tumour continually painful becomes more so from the least fatigue; if this exacerbation of the renal pain is frequent, and if it be accompanied with a complete suppression of purulent urine or with symptoms of inflammation of parts in the vicinity; the operation of nephrotomy, in spite of its difficulties and poor chances, should be resorted to. With greater reason, if fluctuation is perceptible in the lumbar region; if it is evident that pus has collected between the kidney and the sacro-lumbalis muscle; and with still greater reasons, I would say, should a large opening be made without hesitation, after a secondary inflammation of the cellular tissue of this region, or what is much more common, one or more perforations of the kidney distended with pus.

Lafitte and Pouteau have opened abscesses under these circumstances. Not only did they succeed in relieving the patients, but the calculus which had induced the inflammation was found at the bottom of the fistula, and its extraction was followed by a complete cure. I would remark that I think them wrong in denominating the simple opening of the extra-renal abscess, connected with pyelitis calculosa, nephrotomy.

By my invitation, Mr. Velpeau performed with success a similar operation on a patient to whom death without it was inevitable.

The depth of these abscesses, the softening of the neighbouring parts, percolated by the pus, make the delay, in hopes of a *spontaneous* opening, recommended by some, dangerous.

Before treating of the different modes of opening these purulent collections, in order to get at the calculi in the kidney, and to keep open the internal fistula long enough to extract them or allow of their spontaneous exit, I will recount summarily the connection of the tumour with the neighbouring parts.

*Process first.* Incision is, of all the operative processes, that which appears to me applicable to the greater number of cases.

I will suppose a case the most difficult and most rare, that in which it is determined to open the renal pouch without an abscess in the back of the kidney. The patient placed on his well side, the body slightly bent to induce a moderate projection in the lumbar region, the surgeon after close examination of the tumour, which is pressed backwards by the hand of an assistant placed on the front of the tumour, makes an incision from above downwards, three lines from the external margin of the sacro-lumbalis muscle, and parallel to the vertebral column. This first incision commences at the inferior margin of the lower rib, and extends to the crest of the ilium, and includes only the skin and cellular tissue beneath it. By successive incisions we approach the renal tumour, examining the wound with the finger to detect the point where fluctuation is most evident. Having found this, a guarded bistoury is plunged into it, and a fresh incision made before the pus has time to escape. Then with a blunt director or female sound, we examine to see whether the calices and dilated pelvis have been penetrated, or whether we have only penetrated the abscess between the kidney and the square lumbar muscle. In this exploration great care should be observed by the surgeon. I have seen the division of a bridle or the cutting of a vessel followed by considerable hæmorrhage.

If by the index finger of one hand, passed to the bottom of the wound, while the other hand is applied to the anterior surface of the tumour, we perceive the fluctuations of a tumour between the hands, it will be evident that we have but penetrated an extra-renal tumour. Then after carefully sponging out the wound, we should pass the bistoury again into the parts, and penetrate the pelvis and calices by an opening large enough to allow of the exit of pus and the extraction of the calculi.

That which is of most consequence in such a case is to obtain a large opening for the discharge of the pus. This done, the patient should not be fatigued by prolonged explorations, with the hope of extracting calculi. Their situation in the ureter, neck of the pelvis, or in an elongated calix, may make it difficult to discover them; otherwise, should the instrument in dividing the kidney strike one of the branches of the calculus, (they are often branched,) its extraction will be difficult or impossible after a painful exploration. It will be better to wait a few days before attempting the extraction or breaking of the calculi. We should, by proper dressings, preserve a large fistula, which afterwards permits of the extraction of foreign bodies from the kidney.

When the pus collects in the lumbar region just under the skin, the operation presents at first less difficulty. When there is evidently an extra-renal abscess connected with pyelitis calculosa or a renal fistula, the instrument may be propelled at first into the abscess. The opening is afterwards enlarged by a guarded bistoury. Generally, after the discharge of a large quantity of very fœtid pus, the swelling disappears, and the finger introduced into the wound does not meet the renal tumour, but enters the pelvic opening. In this case it is useless to explore much for a calculus; it would be dangerous to introduce in the dark a bistoury into an absorbed and flattened kidney; but for all that the chances of cure are not lost. The kidney may afterwards swell at the bottom of the opening, the calculus may approach from the interior of the extra-renal abscess, and its extraction may be possible through the urinary opening, or by new incisions, as in a case reported by Lafitte.

If, after a pyelitis calculosa and a blind renal-fistula, there is formed a considerable purulent collection in the loins, and if the pus passes behind the peritoneum, and proceeds to pass out at the groin, an incision must be made parallel to the fold of the groin below the crural arch, cutting layer by layer the intervening tissues.

In these cases the patients almost always sink from the suppuration and hectic fever. Tumours formed by the enlarged pelvis and kidney, distended by pus, should never be opened in the anterior part; this would expose the peritoneum or intestine.

*Second process.*—(Incision and puncture.) We are advised not to resort to incision, except to get as near to the renal or extra-renal abscess as possible: to cut only about one and a half inches long: to introduce a trocar into the wound until no resistance is met with: to withdraw the trocar, and allow the pus to pass off through the canula. It is also added, if the collection is very near the skin after withdrawing the canula, to enlarge the wound with a bistoury, but that it is better to retain the canula, or replace it by a gum elastic sound, if the abscess is deep, and if we are not certain that the kidney has contracted adhesions with the neighbouring parts. Finally, Mr. Howship has proposed to provide the styles of the trocar with a gutter, which, by allowing the pus to pass, will indicate to the surgeon when to retract it.

This process, less sure than that which consists in a methodic incision through the parietes of the tumour, has the inconvenience of permitting but an incomplete exploration of the abscess and of the renal pouch.

*Third process.*—(Cauterization and incision.) This process has been applied in cases where the patient would not permit cutting instruments, and in other cases where the abscesses, renal and extra-renal, passed towards the groin or iliac region. This process has the advantage of producing the loss of a certain portion of skin, to induce adhesions around the incision, a useful circumstance in the rare cases where it becomes necessary to cut into the neighbouring intestine, or even where we fear to enter into the cavity of the peritoneum.

This fear, however, need not exist if we cut on the back part of the loins. Besides, cauterization, mild in its effects, is not applicable when the tumour is distended and painful, or when there is reason to fear a spontaneous opening into the peritoneum or into the intestine.

In other cases, incision is much to be preferred, where, for instance, infiltration of pus and urine has taken place into the extra-peritoneal cellular tissue, demanding prompt evacuation. Whether we have recourse to incision, cauterization, or the two modes combined, to open these abscesses; whether we extract or break up the calculus; it is necessary to keep the external opening patulous by means of a tent, in order to allow of the free exit of pus. If, after the operation, we can neither extract nor discover the calculus: and have no better success after seven days, it is best still to retain the fistula open. Calculi have been known to be discharged several years after the operation: and on the other hand, immediate healing of these fistulæ has been followed by serious symptoms, and even death.

I may here recall a remarkable fact recorded by Roonhuysen. After having extracted a stone of some size from an extra-renal abscess of the right kidney, he healed up the wound. The patient enjoyed good health for two entire years, but at the end of that time inflammation was established in the parts. This surgeon, not doubting but that it was caused by another stone, operated again, and extracted one smaller than the first. The wound healed and the patient did well.

In advising to open the tumours formed by a collection of pus in the cavity of the pelvis and calices, or by secondary abscesses about the kidney, I am convinced that these abscesses, unless they open externally spontaneously, are necessarily mortal. The operation itself presents no immediate dangers; the large vessels cannot be involved, especially when there is but one extra-renal abscess; there is no great hæmorrhage to fear, at least in most cases, no danger of involving the peritoneum or intestine. There is in reality against the operation only the difficulty of finding and extracting or breaking the calculi, difficulties which may be postponed, the operation being performed in order to evacuate the pus, and prevent a perforation of the pelvis and calices into the cavity of the peritoneum.

At present, when the structure of these tumours is well known, and the surgeon possesses instruments well calculated to seize and break these stones, the general objections to the operation are much diminished. We may also place in opposition to the unhappy results of the expectant practice, the remarkable and incontestible success obtained by Gaspard Bauhin,* Pouteau,† Lafitte,‡ Labatte,|| Saure,§ Roonhuysen,¶ Colot,** Ledran,†† and many

*A girl born of calculous parents, was attacked by a tumour in the loins, after a total suppression of urine. A surgeon for two months applied, without effect, maturating cataplasms. He observed at last a very hard point in the tumour, on cutting on which he extracted two calculi, with complete success. See Schenck Obs. Medic. Lib. iii. de renibus, Obs. viii.

† Pouteau Melang. de Chir. Lyon 1760, p. 456.

‡ Lafitte, Memoir de l'Acad. Royal. de Chirur. t. ii. p. 233.

§ Labatte, ibid. p. 237. || Saure, ibid. p. 236.

¶ Roonhuysen, Obs. Chirurg. Extraction de deux pierres.

** Colot de la Taille, p. 36 et 40.

†† Ledran, Obs. Chirurg. tom. iii. Obs. lxvi. p. 87.

other practitioners. Doubtless in many of these cases the operation was not exactly nephrotomy; in some the calculus extracted was in an extra-renal abscess, following a fistula of the kidney; and in other cases the calculus not being extracted at the time of the operation, it was found afterwards in the extra-renal abscess, an opening having been made in the pouch of the kidney.

But we must not pretend to perform the operation of nephrotomy—

1st. When we are certain that both kidneys are affected, and probably contain calculi, provided always, that there are no extra-renal abscesses, the perforation of which should neither be neglected or deferred.

2d. When the pus passes freely from the ureter to the bladder, when there is no renal tumour, nor immediate fears of fistula of the kidney, and especially when a favourable condition of the system leads us to suppose that the other kidney has assumed a proportionally increased activity.

3d. When there exist at the same time, incurable lesions of the bladder, prostrate, or of the intestines.

The following case, with the accompanying drawing, exhibits the history, termination, and pathology, of a well marked form of pyelitis calculosa.

*April* 26, 1848. Joseph Ridgway, aged forty years, has had disease of the urinary organs during the last ten years. A merchant by profession; dates the beginning of his disease to exposure by the upsetting of a small boat in the Mississippi, when he contracted a severe fever, ever since which his urinary organs have been diseased. His hair is black, and general appearance, except the emaciation, that of tolerable health. Suffers great pain in the region of the prostate gland. This has always been the case since the commencement of the disease. Discharges blood, mucus, pus and sand, the four layers forming sometimes one fourth of the fluid evacuated. Can scarcely walk; the least jar in stepping downwards produces great pain, and always in the prostate gland. Lies in bed on his left side. The urine on the application of heat and nitric acid, presented *no albuminous flocculi.* Has to evacuate his urine, sometimes, every fifteen minutes. Has been under the care of numerous physicians and surgeons both in this city, and south and west. A distinguished surgeon considered, about three years ago, his disease to be one of the prostate gland, and applied by means of a port caustic the nitrate of silver to the parts. Great irritation resulted from this.

*April* 29. The patient suffers great pain, and is continually pulling the penis, and every five minutes straining over the urinal to evacuate a small quantity of pus, urine and blood, which are very offensive, being of a strong ammoniacal odour. The tongue red, the pulse quick, the skin dry. Directed him to use warm water and thin mucilaginous injections, and apply a large poultice to the perineum.

30*th*. Less pain and difficulty in urination ; continue treatment.

*May* 1. I made an examination of the urethra, bladder and prostate, by introducing a smooth metallic sound through the urethra ; the latter appeared particularly tender near the bladder, but presented no real obstruction to the passage of the instrument. On passing the index finger of the right hand into the rectum, the patient in a standing posture, the curve of the sound could be very distinctly felt in the bladder, and the prostate gland did not appear at all enlarged or particularly tender on pressure. The inflammation of the late exacerbation having apparently subsided, I directed an injection of the acetates of lead and morphia, into the bladder, first washed out with a little tepid water. The patient has been in the habit, in the exacerbations, for the last three years, of drinking freely of Bedford water, which, by increasing the watery portions of the urine, diminishes its acridity and relieves the intense pain. This to be drank pretty freely.

5*th*. Much relieved; less pain; the urine much freer from lateritious and other deposits.

8*th*. Continues to improve under the use of the injection : the urine almost as clear as healthy urine, the sediment now is very dark, and does not exhibit that thick mucous deposit which has heretofore adhered to the urinal like a coat of jelly, when the other portions were poured off. Very little pain, retains the urine three, four or more hours, and at night gets up but once to urinate. Walks about the house very well.

11*th*. Continues in the same favorable condition.

23*d*. Sulphate of iron, gr. viii; sulphate of morphia, gr. iii; pure water, f.℥ iij, were directed as an injection in order to relieve what I conceived to be an inflammation of the bladder. This agreed very well with the parts, and was continued with some unimportant modifications up to

*June 20th*, when another exacerbation came on. The tongue became furred, and the pain in the bladder increased, particularly in the region of the prostate, on which he habitually pressed with one hand, and also pulled the penis; this being the only way in which he could relieve himself. He always lay on the left side, and suspecting from the beginning, disease of the kidney, I found by pressing on the lumbar region, from behind forwards, that tenderness and some pain were experienced in the left kidney; otherwise no pain or uneasiness was experienced in this region. The injections of lead and iron were stopped, and those of slippery elm and flaxseed, with morphia, substituted, with partial relief; morphia and black drop were used by the mouth up to the 30th of this month, when a blister to the sacrum and a mild cathartic moderated the symptoms.

*July* 3. Diarrhœa has set in, and hiccough, with continual pain, invariably in the bladder. Hoffman's anodyne and other things were used in vain for the hiccough.

*7th.* Diarrhœa ceased, hiccough continues; sinking.

*9th.* Died without much pain, apparently from exhaustion. Eighteen hours after death a post mortem examination in the presence of several pupils and physicians, and members of his family, disclosed the following appearances. The left kidney small and reduced to a mere sack, containing in the pelvis and calices a stone, which was the exact shape of these cavities. The ureter enlarged at least six times its normal size, inflamed and tortuous, terminating in a sack or cavity about the size of a hen's egg; which again opened into the bladder in the usual place of the urethral opening. The bladder itself contracted and very much thickened, of a red, verging into, and in the vicinity of the mouth of the urethra over the prostate, a purple colour. The lining membrane of the bladder thickened and covered with a muco-purulent, bloody and sandy matter.

The prostate gland normal in size and consistence. Strong adhesions existed around the left kidney, ureter and the bladder.

The right kidney was hypertrophied and adherent to the lower portion of the duodenum and to the ascending colon; also placed nearly transverse in regard to the spine; highly engorged with blood, but containing no pus or sabulous matter, as was the case in the other kidney. The ureter natural. The plate represents these parts reduced about three-fourths.

The following case,* by my friend Dr. S. Jackson, late of Northumberland, in which the culculi, after forming a tumour in the perineum, passed out by the ulcerative process, shows one of the several directions mentioned by Rayer, in which these bodies find their way to the surface.

"In Februaiy, 1819, I visited Henry Romberger, near Gratz Town, Licken's Valley, thirty-two miles below Northumberland. He laboured under a most distressing dysuria, attended by a very frequent desire to pass water, but, in reality, a sabulous mucus, which came away with great pain and difficulty. Of this he passed great and surprising quantities, but there were no small calculi, *nor could I detect any thing like calculus by sounding.* The patient was about 65 years old, and was greatly broken down by this unremitting distress.

"I gave him a variety of medicines for some time without any relief, unless so far as laudanum procured him better nights. At last we prescribed lime water and castile soap, as used by Dr. Whytt, when, to our very great comfort, as well as his own, he soon began to amend, and ere long he appeared to be perfectly cured.

"After some months he sent for me again, when I found him suffering under his old complaint, with the addition of a large suppurating tumour in the perineum. He told me that immediately after my last visit, he had been persuaded by some of his neighbours, that the medicines I had given him were not in reality curing him, but merely shutting up in his bladder the mischievous matters which had previously come away, in so salutary though painful a manner, and that he ought to take a certain weed in order to discharge them; that having taken the said weed for some time, as advised, his complaint returned, as had been intended, when at last he came to his senses, and perceived the mischief he had done; that a second use of the soap and lime had entirely failed; that he had been suffering most severely, for several days, with the perineal tumour. I recommended him to go on with Whytt's remedies, and to apply a poultice to the perineum.

"In a few days his son came to me in Northumberland, with information that the tumour had discharged a large quantity of

* American Journal of Medical Sciences, August, 1838.

pus mixed with calculi, one of which, as large as a robin's egg, he held in his hand.

"I gave him directions about his father's diet; desired him to return to me, *pro re nata*, and that meanwhile he should use the lime water and soap.

"After some weeks they sent for me to visit the old man once more, when I found him moving about the house clothed in his wife's petticoat, the water coming by drops perfectly clear and natural. He was free from pain, and fast recovering his former health. The fistula in the perineum had entirely healed, and the water was coming by the urethra.

"A cure for this incontinence I considered hopeless, and after promising him some convenience for the reception of his urine, I left him for the last time. He died in a year or two after this period, but I believe his old complaint did not return. There was at least a half a pint of calculi, of various sizes, from that of a robin's egg to that of a pea."

## Figures and Tables

**Figure f1:**